# Probiotics and Vitamin D/Vitamin D Receptor Pathway Interaction: Potential Therapeutic Implications in Inflammatory Bowel Disease

**DOI:** 10.3389/fphar.2021.747856

**Published:** 2021-11-24

**Authors:** Cristiano Pagnini, Maria Carla Di Paolo, Maria Giovanna Graziani, Gianfranco Delle Fave

**Affiliations:** ^1^ Gastroenterologia ed Endoscopia Digestiva, AO S. Giovanni Addolorata, Rome, Italy; ^2^ Gastroenterologia, Università “Sapienza”, Rome, Italy; ^3^ Onlus “S. Andrea”, Rome, Italy

**Keywords:** inflammatory bowel disease, probiotics, vitamin D, vitamin D receptor, homoestasis

## Abstract

Inflammatory bowel diseases (IBD) are chronic conditions of unknown etiology and immunomediated pathogenesis. In the last years, the comprehension of the complex mechanisms involved in the intestinal mucosal homeostasis, and the analysis of the alterations potentially leading to inflammatory pathologic states, has consistently increased. Specifically, the extraordinary impulse in the field of research of the intestinal microbiome has opened the door to the investigation of possible novel approaches to the diagnosis, management and therapeutic applications in IBD. In line with that, administration of probiotic bacteria has been intensely evaluated, leading to much more exciting results in experimental models than in clinical practice. Considering the consistent heterogeneity of the available studies on probiotics, the increased knowledge of the properties of the single bacterial species would ideally lead to unravel potential mechanisms of action that may bring therapeutic applications in specific pathologic condition. Among the relevant molecular pathways for mucosal homeostasis maintenance, the vitamin D/vitamin D receptor (VDR) pathway has been intensely studied in the very last years. In fact, besides osteometabolic functions, the vitamin D exerts important homeostatic effects in the organism at multiple levels, such as immunomodulation, inflammation control, and microbiota regulation, which are likely to play a relevant role in intestinal mucosa protection. In the present review, recent findings about probiotic applications in IBD and mechanisms of action linking vitamin D/VDR pathway to IBD are reported. Available evidence for probiotic effect on vitamin D/VDR are reviewed and potential future application in IBD patients are discussed. At present, many aspects of IBD pathogenesis are still obscure, and current therapeutic options for IBD treatment are at best suboptimal. The increasing comprehension of the different pathways involved in IBD pathogenesis will lead to novel findings ideally leading to potential clinical applications. Microbiota manipulation and vitamin/VDR pathway appear a promising field for future research and therapeutic developments.

## Introduction

In the last decades, virtually every field of human science has been involved and shaked by the effect of the so called “microbiome revolution.” In fact, under the stimulation of novel culture-independent laboratory techniques, that allowed a thorough evaluation of bacterial intestinal species, and thank to an increased comprehension of the molecular mediators of microbiota-intestine interaction, an exponential and intensive interest rising has led to a consistent impulse to this field of research (Rescigno, 2017). Consequently, the idea that the complex eco-system hosted in our gut, collectively comprised in the term “microbiota,” could represent a virtual organ of our organism, with a fundamental role in health maintenance, has more and more decisely taken pace (Marchesi et al., 2016). In line with that, therapeutic manipulation of the microbiota, by means of diet, nutraceuticals, antibiotics, pre- and probiotics has been proposed and investigated in many areas of medicine, with mixed results (Preidis and Versalovic, 2009).

Specifically, the idea of the utilization of beneficial bacterial species for health purposes has been proposed as early as in the beginning of 20th century with the pioneer studies by Elie Methnickov, but it’s at the turn of the new Millennium that the scientific research in that field has consistently grown and expanded (Marchesi et al., 2016). The probiotics, defined by the World Health Organization (WHO) as living organisms with beneficial health effect whether ingested in adequate quantity, has been therefore intensely investigated in experimental models and clinical studies, with more striking results in the former setting comparing with in the latter, mostly due to the extreme dishomogeneity of literature data (Suez et al., 2019). At present, current research on probiotic bacteria is following two main lines. From one side, bacteria with a strong history of empirical utilization and safety data, mainly from Lactobacilli and Bifidobacteria genera, have been rigorously and carefully investigated in pre-clinical and clinical studies, in order to propose and solidly support clinical utilization in specific situations (Kleerebezem and Vaughan, 2009). On the other hand, by means of an accurate microbiota composition analysis, difference between health subjects and patients with different diseases has been characterized, with the final ideal goal to identify bacterial species of particular relevance for the pathologic condition, potentially useful as novel probiotic bacteria (“next generation probiotics”) to supplement for therapeutic purposes (O'Toole et al., 2017). Regardless the research approach and notwithstanding the actual flaws for an evidence-based utilization of probiotics, the clearest concept emerged is that probiotics are not the same, but many molecular and therefore potential clinical effect are often species-specific and not generally extendable (McFarland et al., 2018). Accordingly, the generic term “probiotic” has nowadays lost its sense, considering that, at present, and many more in the future, the identification of specific molecular properties of well characterized bacterial species, and the correct and aimed positioning in a specific clinical setting, it’s most probably the key to the implementation of probiotics utilization as a therapeutic option in medicine.

Among the infective and inflammatory pathologies where probiotics’ application has been investigated, inflammatory bowel diseases (IBD) still represent one of the most promising and yet debated (Ghouri et al., 2014). IBD are a group of diseases, whose two main forms are represented by ulcerative colitis (UC) and Crohn’s disease (CD), clinically characterized by intermittent/recurrent symptoms of active disease (abdominal pain, diarrhea, bloody stool) and remittent phases. Even though these two entities share pathogenetic similarities, they present peculiar morphological and clinical features. UC is characterized by a chronic inflammation of the superficial layer of the colonic mucosa, initiating in the rectum and with a variable proximal extension, while in CD the mucosal inflammation is transmural and may affect virtually every segment of the GI tract with skip lesions, and may be characterized by prevalence of inflammation or complications such as stenosis and fistulas (Abraham and Cho, 2009). Among available pharmacological treatments there are mesalamine, corticosteroids, antibiotics, immunosuppressant and biologic drugs, with the latter representing the mainstay of treatment for moderate-severe disease (Lamb et al., 2019). Despite conventional and immunomodulatory therapy, still many patients do not respond adequately, so that the research and the development of novel pathways involved in disease occurrence, to be targeted for therapeutic purposes, are largely needed. Among possible involved molecular pathways, in very recent years the vitamin D/vitamin D receptor (VDR) interaction has been consistently proposed (Kellermann et al., 2020). In fact, besides its well characterized role in bone metabolism, vitamin D has been recently highlighted as an important molecular mediator for intestinal homeostasis, due to important immunomodulatory and anti-inflammatory effect (Del Pinto et al., 2017). Since bi-univocal links between microbiota and vitamin D has been hypothesized, the idea of a potential therapeutic application of probiotic bacteria and vitamin D in IBD patients appears more than attractive.

In the present narrative review we intended to critically analyse pre-clinical and clinical available data on potential influence of probiotic and vitamin D pathway interaction in IBD patients. The concomitant use of probiotic and vitamin D could be helpful in IBD patients both for the single potential positive effect on intestinal inflammation that probiotics and vitamin D may exert singularly, and for a real molecular interaction with a reciprocal amplification of effect. Therefore, we briefly summarized the experimental and clinical data for probiotic and vitamin D efficacy in IBD separately, and then we explored the possible interaction at molecular level and the clinical effect of probiotic/vitamin D concomitant administration.

## Probiotics in IBD: Potential Mechanism of Action and Clinical Evidence

Evidence for a microbial influence in IBD onset and/or development comes from initial observations from germ-free animals and in patients with fecal diversion, indicating a negative role of intestinal bacteria (Rutgeerts et al., 1991; Taurog et al., 1994). More recent data suggest that an altered balance between protective and pathogenic bacteria occurs in IBD patients (“dysbiosis”), potentially contributing to the initiation and progression of a deregulated chronic inflammation (Caruso et al., 2020). Indeed, a consistent set of experimental and pre-clinical data indicate potential mechanisms of action by which specific probiotic bacteria may exert a beneficial effect on chronic intestinal inflammation (Ciorba, 2012). In fact, probiotics may contrast the dysbiosis by reducing pathogenic bacteria and stimulating beneficial ones, such as butirrate-producing bacteria (Markowiak-Kopec and Slizewska, 2020). Moreover, they may temporary colonize the intestinal mucosa and directly interact with specific receptors of the innate immune system, namely the pattern recognition receptors - PRR (i.e., nucleotide-binding oligomerization domain - NOD and toll-like receptors - TLRs), thus exerting an immunomodulatory effect (Bermudez-Brito et al., 2012). As a consequence, epithelial functions are enhanced, with stimulation of cytoprotective factors, improving of epithelial cells survival, stimulation of mucus and anti-bacteria molecules production, reduction of intestinal permeability (Ohland and Macnaughton, 2010). The increase of the intestinal barrier efficacy reduces the antigen load to the sub-mucosal compartment, and for that reason, and for a direct effect of probiotics on dendritic cells and lymphocytes, adaptive pro-inflammatory immune response is prevented and reduced, with a reduction of pro-inflammatory cytokines (i.e., TNF, IFN, IL-17) and a stimulation of regulative mediators (i.e., IL-10, TGFb, IL-4) (Pagnini et al., 2013). Unfortunately, the impressive experimental data have not be followed so far by convincing clinical results, and clinical trials in IBD patients have been characterized by a dramatic dishomogeneity in terms of probiotic used, doses and duration of the therapeutic schemes, inclusion criteria and end-points investigated, so that clear evidences are far from being depicted. In fact, attempts to synthetize clinical data into meta-analysis yielded to inconsistent results (Limketkai et al., 2020a; Iheozor-Ejiofor et al., 2020; Kaur et al., 2020). Nonetheless, utilization of *E. coli* Nissle 1917 for remission maintenance in UC patients and of VSL#3 probiotic mixture in pouchitis is indicated as possible options in international guidelines (Harbord et al., 2017; Su et al., 2020), suggesting that well designed clinical trials would ideally expand utilization of more probiotic species in specific IBD setting and indications. Indeed, a very recent study brilliantly highlighted that the variable results of probiotics in human studies may be related to two conceptual shortcomings: first, the fact that most studies rely on fecal, rather than mucosal, probiotic concentration as a marker of colonization, and second, the lack of appropriate investigation of the subjects’ microbiota before probiotic administration, since different composition has been found to be related to a “permissive” or “resistant” phenotype to exogenous bacteria administration (Zmora et al., 2018).

### Vitamin D/VDR and Immune System Regulation in IBD

Vitamin D is a fat soluble secosteroid hormone that can be assumed in the diet in two forms: vitamin D2 (ergocalciferol), present in mushrooms and vegetables, and vitamin D3 (colecalciferol), in fish and meet. The alimentary source is substantially scarce, and vitamin D3 is endogenously synthetized in the skin for the transformation by the UV light of the cholesterol precursor 7-dehydrocholesterol in pre-vitamin D3 and then in vitamin D3. In the blood stream, vitamin D3 and D2 are converted by a double hydroxylation process in the liver, by the enzyme 25-hydroxylase (CYP2R1) in 25 hydroxyvitamin D (25(OH)D), and in the kidney, by the enzyme 1-α-hydroxylase (CYP27B1), into its active form, 1,25-dihydroxyvitamin D (1,25(OH)2D or calcitriol). VDR is a single aminoacidic chain polypeptide of the nuclear receptors superfamily, and it is widely and differently expressed in many tissues, including intestinal mucosa and immune cells (Pike et al., 2017). The binding of 1,25(OH)2D to VDR in the cytoplasm of the cell, with the heterodimerization with the retinoid X receptor (RXR), determines the translocation of the complex to the nucleus and the binding to vitamin D response elements (VDREs), with stimulation and/or suppression of gene transcription (Pagnini et al., 2021). The biologic action of vitamin D/VDR signalling, initially characterized in the bone metabolism, is pleiotropic, and the correct functioning of this pathways has a paramount role for homeostasis maintenance at several levels. Multiple molecular effects may have a positive role in preventing and ameliorating chronic intestinal inflammation in IBD patients, and in particular the enforcement of intestinal barrier, the immunomodulation, and the microbiota modulation (Kellermann et al., 2020). In fact, experimental data indicate that vitamin D/VDR signalling stimulates functionality of tight junction proteins. VDR knockout and vitamin D-deficient mice showed epithelial barrier impairment with hyperfunction of claudin-2, and increased susceptibility to invasive bacteria colonization and colitis (Assa et al., 2015; Zhang et al., 2019). Vitamin D supplementation showed beneficial in Dextran sulphate sodium (DSS) model of colitis, by preserving the expression of E-cadherin, claudin, and zonula occludens in Caco-2 cells (Zhao et al., 2012). At intestinal mucosal level, vitamin D/VDR interaction display immunoregulatory effect, with a global stimulation of innate defence and regulation of pro-inflammatory mediators of the acquired compartment of immunity (Kellermann et al., 2020). In fact, vitamin D induces a TLR2/1-dependent activation of cAMP and beta-defensin 2 expression in monocytes and macrophages, with an increased anti-microbial function, and a vitamin D deficient diet or a lack of VDR can determine impaired anti-bacterial activities of epithelial cells and increased inflammation (Liu et al., 2009; Wang et al., 2010). Experimental data demonstrate that vitamin D stimulates autophagy, that is an essential innate immune physiological mechanism by which potentially harmful antigens are cleared at the mucosal compartment, thus preventing gut inflammation and dysbiosis (Wang et al., 2010). Considering adaptive immunity, many data indicate that vitamin D inhibits Th1, Th17 cells, and DCs differentiation and promoting Treg cells, with a reduced production of pro-inflammatory cytokines [i.e., IL-17A, TNF-alpha, IL-6, and interferon-gamma (IFN-γ)] (Kamen and Tangpricha, 2010; Wang et al., 2010). The effect on innate immunity is probably the key for the modulation of intestinal microbiota by vitamin D. In a recent study in a mouse model with a lack of expression of VDR in Paneth cells, Lu et al. elegantly demonstrated that VDR signalling is essential for bacteria recognition, pathogens clearance and dysbiosis prevention (Lu et al., 2021). In fact, vitamin D administration has demonstrate to influence microbiota composition in mice models and human studies, potentially preventing or correcting dysbiosis (Shang and Sun, 2017). In particular, a recent meta-analysis of fourteen studies confirmed a regulatory effect of vitamin D administration on intestinal microbiota composition, even though with mixed results (Waterhouse et al., 2019), and even studies investigating microbiota modification due to vitamin D in IBD patients showed conflicting results (Garg et al., 2018; Schaffler et al., 2018; Soltys et al., 2020). Considering clinical data, low vitamin D status has been found to be associated with a higher IBD risk and a recent meta-analysis, including nearly 1,900 subjects, showed that IBD patients had a 64% increased risk of vitamin D deficiency comparing with controls (Del Pinto et al., 2015). Even more recently, a meta-analysis including a total of 8,316 IBD patients (3115 UC, 5201 CD), showed that low 25(OH)D level was linked to higher risk of disease activity, mucosal inflammation, low quality of life (QOL) scores, and clinical relapse (Gubatan et al., 2019). Considering the low vitamin D levels and IBD occurrence/severity, the crucial question of whether it represents a cause or an effect remains still unsolved, even though recent observational studies examining vitamin D levels prior to the diagnosis of IBD seem to support the latter hypothesis (Opstelten et al., 2018; Limketkai et al., 2020b). Interventional studies investigating the effect of vitamin D supplementation in IBD patients are still preliminary and no clear evidence exists, but a recent meta-analysis of 18 studies, with a total of 908 IBD patients, indicated that vitamin D supplement significantly improved the 25(OH)D blood levels and, in seven trials, determined a consistent relapse rate reduction comparing with untreated patients (Li et al., 2018). Indeed, for the established role of vitamin D for the bone health and the high incidence of deficiency in IBD patients, periodic check and correction of insufficient levels is advisable in such patients, even though the administration for immunomodulatory purposes remains, at present, only a fascinating suggestion (Myint et al., 2020). Moreover, since the correction in deficient IBD patients appears rational and indicated, the beneficial effect of vitamin D supplement in patients with normal serum level is not straightforward and probably needs further investigation.

## Probiotics Plus Vitamin D: Evidence for a Synergic Effect

### Molecular Interaction

**FIGURE 1:**
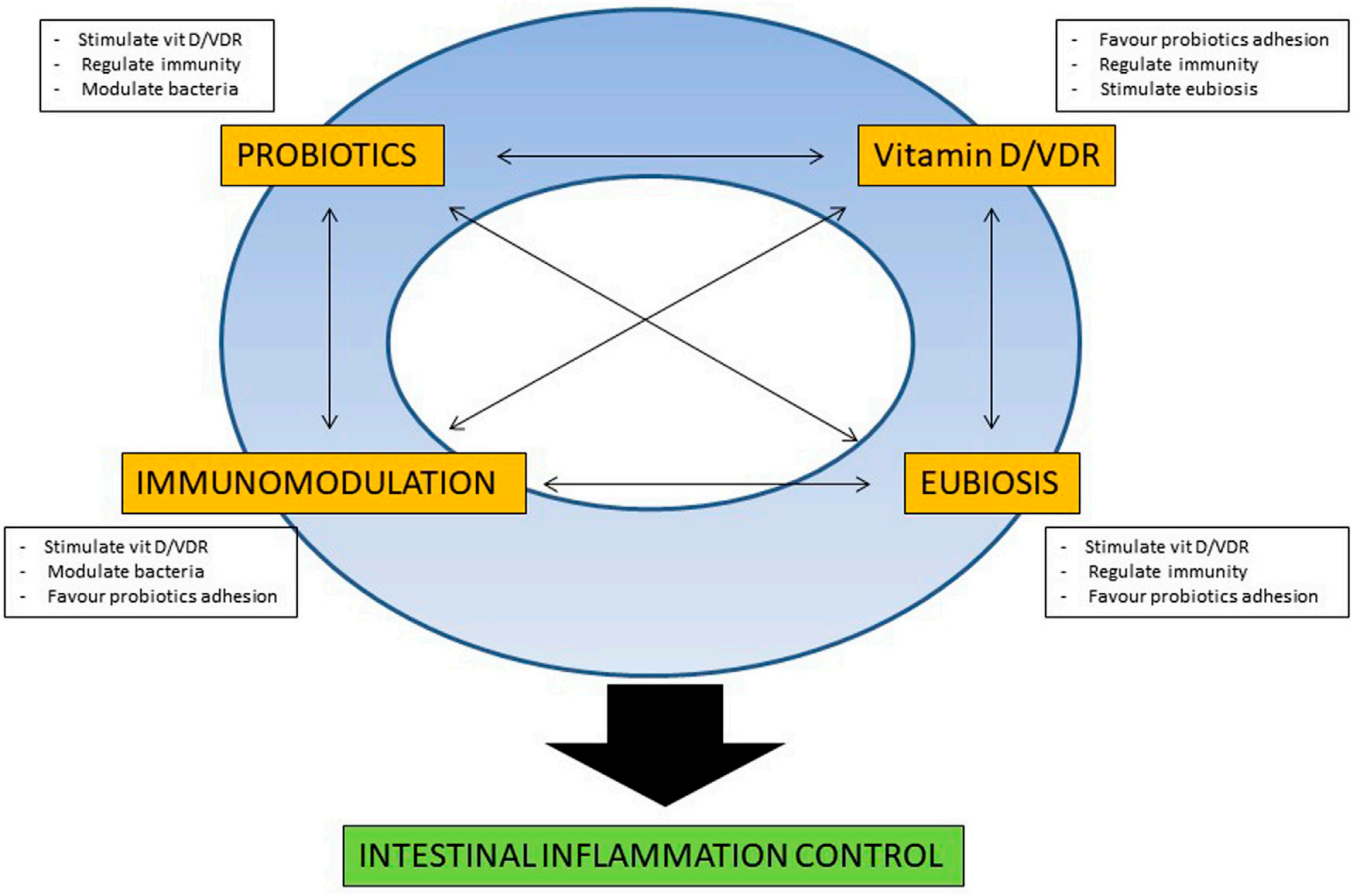
Schematic representation of the possible reciprocal molecular interactions between probiotics and vitamin D for intestinal mucosal homeostasis. For a detailed explanation refer to the text.

Besides the aforementioned beneficial effect that probiotics and vitamin D may singularly exert in IBD patients, early experimental data are suggesting a possible direct interaction between those two nutraceuticals, that may confer increased anti-inflammatory effect in the intestinal mucosa. In fact, studies in VDR knock-out (KO) mice have shown a defective autophagy and presence of dysbiosis, with reduction of Lactobacilli and Bacteroidetes species, comparing with wild-type mice (Ooi et al., 2013). In experimental model of colitis, supplementation of butyrate stimulate VDR genetic expression and protein production, with amelioration of the colonic inflammation (Wu et al., 2015b), even though the exact contribution of the VDR pathway for the anti-inflammatory effect of butyrate is not completely elucidated, considering the concomitant activation of the cell surface G-protein coupled receptors (GPCRs) such as GPR41, GPR43, and GPR109A, potentially involved for the immunomodulatory effect of butyrate in intestinal mucosa (Parada Venegas et al., 2019). Moreover, recent studies demonstrated that VDR functioning pathway is necessary for probiotics protection against colitis. In an elegant study, Wu et al. demonstrated that *Lactobacillus rhamnosus* GG and *Lactobacillus plantarum* stimulated VDR expression and activity in different cell lines, and that the administration of the two probiotic bacteria had a protective effect against Salmonella-induced colitis only in wild-type mice with intact functioning of the VDR pathway, while that protective effect was completely abrogated in VDR knock-out (KO) mice (Wu et al., 2015a). In addition, further experimental data demonstrated that probiotics stimulate VDR expression and activity. In the trinitrobenzene sulfonic acid (TNBS) inflammation-cancerogenesis model, the administration of the multiple probiotic compound VSL#3 stimulated VDR expression (together with angiostatin and alkaline sphingomyelinase), thus delaying the inflammatory mediated transition to dysplasia and cancer (Appleyard et al., 2011). The same multi-species probiotic product has shown to induce expression and modulate activity of VDR and other nuclear receptors, in an animal model of genetic dyslipidemia, with a reduction of insulin resistance in liver and adipose tissues and protection against development of steatohepatitis and atherosclerosis (Mencarelli et al., 2012). Early administration of *Lactobacillus casei* BL23 in larval zebrafish positively influenced growth, immune system development and survival, by means of induction of genes with different involvement in homeostasis, among which VDR-α (Qin et al., 2018). As a further confirmation of the strain-specificity properties of probiotic bacteria, among six Lactobacillus strains tested, only *L. plantarum* significantly induced VDR expression in HT-29 MTX cells (Raveschot et al., 2020), even though the association between increased expression of VDR and its activity is still not fully demonstrated. Besides the effect on VDR, some clinical and experimental data indicate that probiotic bacteria may increase vitamin D levels. In a post-hoc analysis of a randomized controlled trial investigating the cholesterol-lowering efficacy of the bile salt hydrolase active *Lactobacillus reuteri* NCIMB 30242, surprisingly, the probiotic bacteria did not impaire the absorption of fat-soluble vitamins, and yet increased the mean circulating level of 25-Hydroxyvitamin D, after 9 weeks of administration (Jones et al., 2013). In clinical studies including patients after bariatric surgery, administration of a multiple probiotic compound, from 4 weeks prior to 12 weeks after surgery, increased 25-OH Vitamin D serum level in patients undergoing One Anastomosis Gastric Bypass- Mini Gastric Bypass (Karbaschian et al., 2018), and the same effect was observed for an association of *Lactobacillus acidophilus* NCFM and Bifidobacterium lactis Bi-07, administered for 3 months after Roux-en-Y Gastric Bypass (Ramos et al., 2021). In a computational modeling framework analysis, prebiotic stimulates pro-vitamin D3 by means of an increased production of lactate by stimulated Lactobacilli (Gokhale and Bhaduri, 2019). Although the exact molecular mechanism for the increased vitamin D by probiotics remains to be elucidated, possible factors are the increased absorption at intestinal level, mediated by increased ion concentration and lower pH, the increased substrate concentration, given by the lactate produced by the probiotic bacteria, and the activity stimulation of key enzymes of the vitamin D pathway, such as hepatic 25-hydroxylase or hepatic 3-hydroxy-3-methyl-glutaryl-coenzyme A reductase (Hollander et al., 1978; Yavuz et al., 2009). Therefore, considering experimental data, a hypothetical model for probiotic/vitamin D interaction for their beneficial effect in IBD patients could be drawn, as represented in Figure 1. In a virtual circle with multiple reciprocal interactions, specific probiotic bacteria may increase circulating vitamin D levels and stimulate the mucosal expression and activity of VDR, that in turn may exert immunomodulation of the mucosal immunity, with an enforcement of innate and anti-bacterial defences and a reduction of Th1 polarized cytokines, with a global anti-inflammatory mucosal effect. The stimulation of the innate response contributes to positively regulate the intestinal microbiota and to resolve or prevent dysbiosis, further favouring temporary colonization of administered probiotic bacteria and the stimulation of proliferation of butyrate-producing bacteria, with a consequent activation of vitamin/VDR pathway in a looping manner.

### Clinical Data

Despite mounting data on potential biological interaction between vitamin D and probiotics, clinical data are still at the beginning. To date, ten randomized clinical trials (RCTs) (Savino et al., 2015; Tazzyman et al., 2015; Miraglia Del Giudice et al., 2016; Jafarnejad et al., 2017; Raygan et al., 2018; Ghaderi et al., 2019; Jamilian et al., 2019; Ostadmohammadi et al., 2019; Hajipoor et al., 2021; Morvaridzadeh et al., 2021), investigating the application of co-administration of vitamin D and probiotics, has been published (Table 1), seven of which have been included in a recent systematic review (Abboud et al., 2020). No clinical trial investigated so far the simultaneous application of probiotics and vitamin D in IBD patients. Waiting for clinical data, utilization of those nutraceuticals appears rational and may already be proposed as a supportive treatment in induction and maintenance of remission, as an ancillary therapy to the evidence-based treatments currently approved and available. In fact, considering the safety profile and the rational for their utilization, they may contribute to increase treatment efficacy and improve the management of IBD patients. Encouraging clinical data comes from different settings, and nearly all the published studies demonstrated a beneficial effect of probiotics and vitamin D co-administration. Nonetheless, results need to be taken with great caution, and clinical data in this field have to be considered preliminary. In fact, a consistent dishomogeneity exists in published studies, since trial designs, therapeutic schemes, probiotic species, probiotics/vitamin D doses, duration of treatments, clinical settings, and sample sizes profoundly differ. Among published trials, only one investigated a gastroenterological condition, namely irritable bowel syndrome (IBS) (Tazzyman et al., 2015). No significant difference in symptoms was observed between patients who had co-supplementation with probiotics and vitamin D, compared with those who had vitamin D alone, or placebo. However, this study had a limited sample size and a limited duration of follow-up, and presented a consistent placebo effect, which may be due to different sun exposure between the investigated groups. Among the tested pathologic conditions, particularly positive results of vitamin D/probiotics administration has been observed in metabolic disorders, potentially representing a promising path for future research.

**TABLE 1:** Randomized clinical trials (RCTs) investigating the effect of co-administration of probiotics and vitamin D in different clinical conditions; no trial, at present, evaluated the effect of probiotic plus vitamin D in IBD patients.

Study (first author, year)	Disease	N	Vitamin D dose	Probiotic species	Comparator	Outcome
Ghaderi (2019)	Schizofrenia	60	50,000 IU/2 weeks	*L. acidophilus*, *B. bifidum*, *L. reuteri*, *L. fermentum*	Placebo	Beneficial
Jafarnejad (2017)	Osteopenia	50	200 IU/day	*L. casei*, *B. longum*, *L. acidophilus*, *L. rhamnosus*, *L. bulgaricus*, *B. breve*, *S. thermophilus*	Vitamin D alone	Some molecular difference but no effect on BMD
Jamilian (2019)	Gestational diabetes	87	50,000 IU/2 weeks	*L. acidophilus*, *B. bifidum*, *L. reuteri*, *L. fermentum*	Probiotic alone; placebo	Beneficial
Ostadmohammadi (2019)	Polycystic ovary syndrome	60	50,000 IU/2 weeks	*L. acidophilus*, *B. bifidum*, *L. reuteri*, *L. fermentum*	Placebo	Beneficial on mental health but no effect on other parameters
Raygan (2018)	Type 2 diabetes	60	50,000 IU/2 weeks	*L. acidophilus*, *B. bifidum*, *L. reuteri*, *L. fermentum*	Placebo	Beneficial on mental health, glycemic level, HDL, CRP but no effect on other metabolic profiles and hypertension
Savino (2015)	Infantile colic in newborns	105	400 IU/day	*L. reuteri* DSM 17938	Vitamin D alone	Beneficial
Tazzyman (2015)	IBS	51	3,000 IU/day	*L. acidophilus*, CUL 60, CUL 21, *B. bifidum* CUL 20, *B. animalis* sub. Lactis CUL 34	Vitamin D alone+placebo; placebo+placebo	No effect
Miraglia Del Giudice M (2016)	Asmatic allergic children	32	400 IU/day	*L. reuteri* DSM 17938	Placebo	Beneficial
Hajipoor S (2021)	Obese	140	1,000 IU/day	*L. acidophilus* La-B5, *B. lactis* Bb-12	1)Plain yogurt, 2)yogurt+probiotics alone, 3)yogurt+vitamin D alone	No difference in lipid profile, anthropometric indices
Morvaridzadeh M (2021)	NAFLD	104	1,000 IU/day	*S. thermophilus*, *L. bulgaricus*, *L. acidophilus* La-5, *B. lactis* Bb-12	Plain yogurt	Beneficial on 25(OH)D3 level, no effect on blood sugars and antropometric parameters

L. , – Lactobacillus, B. – Bifidobacterium, IBS – irritable bowel syndrome, NAFLD – non-alcoholic fatty liver disease.

## Future Perspectives and Conclusion

Despite pre-clinical data for a possible interaction of vitamin D/VDR pathway and probiotic administration in ameliorating intestinal inflammation, clinical studies are still to come. Considering the encouraging clinical data from other clinical settings, this therapeutic option appears intriguing and promising and deserve future investigation. In order to design reliable trials, flaws emerged from pre-clinical and clinical studies in probiotics and vitamin D application in IBD needs to be taken into account and carefully addressed. First, the choice of the probiotic bacteria appears to be relevant, considering that beneficial properties may differ even at strain level. A well studied bacterial species, with solid safety data and documented anti-inflammatory effect in the intestinal mucosa, could most probably lead to better results. Moreover, pre- and post-interventional assessment of microbiota quali-quantitative composition, together with the verification of temporary mucosal colonization of the supplemented probiotic, by means of genomic-based techniques, may provide further insights into potential mechanism of action of nutraceuticals, pre-selection of patients, and identification of potential markers for efficacy evaluation. Considering the high rate of vitamin D deficiency, and the lack of specific target levels for IBD patients, assessment of pre- and post-interventional blood levels, and evaluation of VDR mucosal expression, could help in identifying surrogate markers to pre-stratify patients and to monitor and guide nutraceutical supplementation modalities. In this regard, the possible presence of polymorphism of VDR genes (namely, TaqI and FokI), described in up to 20% of IBD patients, that may influence VDR functionality and therefore potentially reduce the response to vitamin D administration (Xue et al., 2013), need to be probably assessed. Finally, considering the variability of the clinical pictures that fall under the term of “IBD,” it is necessary to design interventional studies in specific restricted homogeneous clinical condition, as for example UC patients with proctosigmoiditis or CD patients with inflammatory phenotype and exclusive ileal localization. Moreover, possible confounding factors, such as diet, sun exposure, metabolic status, co-morbidities, and drug utilization, should be carefully assessed and standardized. In conclusion, vitamin D/probiotics co-administration appears a rational and attracting therapeutic option in IBD patients, but clinical data do not exist yet. The appropriate design of reliable trials will help to evaluate the potential efficacy, identify specific conditions and administration modalities, that would support and propose the contemporary supplement of vitamin D and probiotic in clinical practice for IBD patients.
